# Evaluation of Trends in the Use of Complementary and Alternative Medicine in Health Centers in Khorramabad (West of Iran)

**DOI:** 10.5539/gjhs.v8n2p72

**Published:** 2015-06-05

**Authors:** Khatereh Anbari, Mohammadreza Gholami

**Affiliations:** 1 School of Medicine, Lorestan University of Medical Sciences, Khorramabad, Iran

**Keywords:** complementary medicine, traditional medicine, health, treatment centers

## Abstract

**Aim:**

To determine the use of most popular forms of complementary and alternative medicine(CAM), sociodemographic characteristics of CAM users, and communication between CAM users and their physicians by adult Iranian in Khorramabad city.

**Methods:**

This cross-sectional study was carried out on clients who were at least 15 years in age referring to health centers and hospitals in Khorramabad town in 2014. A multi-part questionnaire was used to gather information. The demographic data and details regarding usage (number of times and underlying reasons) of different kinds of complementary and traditional medicine in the past were gathered using a questionnaire.

**Results:**

In this study 790 subjects were surveyed using the questionnaire. The mean age of the participant was 38.9 years. 79.8% of the subjects had used at least one of the methods of complementary medicine. Among the participants, 58.2% had used at least one of the complementary medicines in the previous year. Herbal medicine and prayers treatment had the highest use with 69.2% and 37.2%, respectively. Concerns of the side effects of medical therapy, beliefs in less risky being and fewer side effects of complementary medicine, Dissatisfaction of General Practitioners, the increase of being-well feelings in physical conditions, and were among the most important reasons of inclination towards such treatment methods.

**Conclusion:**

The analysis of using complementary medicine among people is the first step for planning proper use of the beneficial methods of complementary medicine and the prevention of inefficient and harmful methods in this respect.

## 1. Introduction

Complementary and alternative medicine (CAM) is used for medical treatment that is not part of the conventional, science-based healthcare (Ock Choi, Ka, Lee, Chun, & Huh, 2009; Saad, Azaizeh, & Said, 2005; Xue et al., 2007). These practices are widely used in conventional medicine to treat patients and prevent disease in healthy individuals (Ock et al., 2009, Saad et al., 2005, Xue, Zhang, Lin, Da Costa, & Story, 2007). Complementary medicine includes medical procedures and interventions that are not widely taught in medical schools and hospitals are usually not available (Ock et al., 2009, Saad et al., 2005, Xue et al., 2007). Today, complementary medicine alongside conventional medicine is widely used to treat and restore health to the sick and prevention of disease in healthy individuals (Ock et al., 2009, Saad et al., 2005, Xue et al., 2007). In recent years the use of various methods of alternative medicine has further upside in many countries, including developing countries. The use of complementary medicine in the world has been growing interest e.g. Belgium (66-75%), France (49%), Australia (46%), America (34%), UK (33%), Germany (20-30%) and Netherland (18%) (Zollaman & Vickers, 1999). Studies show that people who have difficulty in using conventional medicine or are dissatisfied with the quality of traditional medicine are more likely to use CAM (Ritchie et al., 2005). Excessive use members of society without awareness of the potential dangers of these methods are their main concern. Mistake in dosage or route of administration and deprived of appropriate treatment effects are irreversible. Cause of CAM use has been proposed such as frustration of conventional therapies, economic problems and deprived of conventional medicine, cheaper and the availability of complementary medicine, inability to pay current treatments and convinced that providers of complementary medicine therapies listeners are better than (Sedighi et al., 2002). The aim of this study was to investigate the use of most popular forms of complementary and alternative medicine (CAM) by adult Iranian in Khorramabad city, sociodemographic characteristics of CAM users, and communication between CAM users and their physicians.

## 2. Methods

This cross-sectional study was performed in Khorramabad City in 2014. Inclusion criteria for the study have been included people living in the city of Khorramabad, age of 15 years and no cognitive and emotional problems. The multi-stage sampling was used. First of all, health centers in the city of Khorramabad have been listed (N=19) than 10 health centers were randomly selected. People referred to these centers were recruited accessible sample. Sampling was done for the various services of people who were referred to health centers. A multi-part questionnaire was used to collect data. The questionnaire included demographic characteristics of individuals and their health insurance status. In the second part about using any of a variety of complementary medicine was questioning in a lifetime and in the past year. The third part of the questionnaire contained questions on the subjects of interest to the methods of complementary medicine. Degree of each question importance was classified according to Taipei scale. To determine the validity of questionnaire was used the opinions of experts in traditional medicine, a clinical psychologist and an expert in social medicine. To determine the reliability of the method was used Cronbach’s alpha (α=0.78). To develop the questionnaire a number of resources have been used such as traditional medicine books and papers published in the field of traditional medicine and complementary (Moradi Lakeh, Ramezani, & Ansari, 2008; Sedighi, Maftoon, & Moshrefi, 2002).

### 2.1 Statistical Analysis

The data was analyzed using the SPSS software and the proportion and percentages were used to describe the data.

## 3. Results

In this study, a total of 790 subjects were asked questions. The mean age of participants was 38.9 ± 9.38 years. Youngest participant was 15 years old and the oldest 81 years old. Information on demographic characteristics of participants is given in Table 1. Participants (79.8%) were used at least one of the subjects CAM and participants (58.2%) were used at least one of CAM during the past year. Medicinal herbs, prayer, cupping and the hydrotherapy had the highest consumption with 69.2%, 37.2%, 16.2% and 13.9%, respectively (Table 2). Chiropractic techniques (8.0%), homeopathy (9.0%) Hypnosis (4.1%) had the lowest uses (Table 2). Cause of the use of complementary medicine approaches from the perspective of the subjects in order of importance are; concerns of the common side effects of medications commonly, believed to reduce the risk of complications and less of complementary medicine, dissatisfaction of general physicians, increase the sense of improving the health status and fear of recurrence of the underlying disease (Table 3).

**Table 1:** Distribution of demographic characteristics the studied population

Frequency (percent)	Frequency (number)	Variable type
Age:		
**29-15**	**245**	**(31)**
**44-30**	**271**	**(34/3)**
**59-45**	**204**	**(25/8)**
**60 ≥**	**70**	**(8/9)**

Gender:		
Male	**352**	**(44/6)**
Female	**438**	**(55/4)**

Education:		
Uneducated	**34**	**(4/3)**
Middle school Diploma	**118**	**(14/9)**
Graduate student	**297**	**(3/6)**

Job:		

Unemployed	**129**	**(16/3)**
Worker	**41**	**(5/2)**
Employee	**150**	**(19)**
Self-employment	**119**	**(15/1)**
Housekeeper	**282**	**(35/7)**
Military	**17**	**(2/2)**
Farmer	**10**	**(1/3)**
Student	**42**	**(5/3)**

Residence:		
Town	**630**	**(79/7)**
Rural	**160**	**(20/3)**

**Table 2:** Frequency of use of complementary and alternative medicine methods in the subjects

Type of procedure	Lifetime history Number (percentage)	Use in recent years Number (percentage)
Herbal medicines	**547 (69/2)**	**298 (37/7)**
Cupping	**128 (16/2)**	**38 (4/8)**
Acupuncture	42 (5/3)	**17 (2/15)**
Massage therapy	**53 (6/7)**	**11 (1/4)**
Praying therapy	**294 (37/2)**	**185 (23/4)**
Energy therapy	24 (3)	**5 (0/63)**
Hypnotic	**11 (1/4)**	**2 (0/25)**
Hydrotherapy	110 (13/9)	**42 (5/31)**
Yoga or Meditation	**29 (3/7)**	**9 (1/13)**
Leech therapy	27 (3/4)	**13 (1/64)**
Sand therapy	27 (3/4)	**9 (1/13)**
Homeopathy	**7 (0/9)**	**3 (0/37)**
Bee sting	**15 (1/9)**	**5 (0/63)**
Chiropractic	**6 (0/8)**	**4 (0/5)**
Stone therapy	19 (2/4)	**9 (1/13)**
Music therapy	**69 (8/7)**	**29 (3/67)**
Acupressure	**40 (5/1)**	**14 (1/77)**

**Table 3:** Cause of the use of CAM in the study

Causes of Tendency	The degree of importance of each factor			Mean±SD
	High Number (percentage)	Middle Number (percentage)	Low Number (percentage)	
These treatments are thought to boost the immune	167(21/1)	291(36/8)	322(42)	1/79±0/70
system Economic problems and the deprived of	181(22/9)	236(29/9)	373(47/2)	1/75±0/8
conventional medicine Spend more time on	154(19/5)	241(30/5)	395(50)	1/69±0/77
traditional therapists The immediate effects of CAM	137(17/3)	284(35/9)	369(46/7)	1/70±0/74
Dissatisfaction of general physicians	247(31/3)	314(39/7)	229(29)	2/02±0/77
Lack of communication skills of physicians and the inadequate information about the disease	195(24/7)	218(27/6)	377(47/7)	1/76±0/81
Concerns about the side effects of conventional medicine	366(46/3)	282(35/7)	142(18)	2/28±0/75
Fear of recurrence	216(27/3)	222(28/1)	352(44/6)	1/82±0/83
Low risk of complications and less these methods	342(43/3)	255(32/3)	193(24/4)	2/18±0/8
Increase the sense of improving the health status	203(25/7)	259(32/8)	328(41/5)	1/84±0/8
Cheaper in comparison with conventional medical treatments	177(22/4)	255(32/3)	358(45/3)	1/77±0/79
Failure to respond adequately to conventional medical treatments	166(21)	283(35/8)	341(43/2)	1/77±0/77
Disappointment conventional therapies	185(23/4)	275(34/8)	330(41/8)	1/81±0/78

## 4. Discussion

Results suggest that the population studied, more than 3/4, at least one of a variety of methods are used CAM in their lifetime and half of the above mentioned methods were used during the last year. Tehrani-Banihashemi et al., showed that the prevalence of CAM use annual rate were 52/5% and prevalence of CAM use at over lifetime were reported 66/3% (Tehrani-Banihashemi, Asgharifard, Haghdoost, Barghamdi, & Mohammadhosseini, 2008). The other objective of the present study was to investigate the causes of using CAM in the population of study. About the reasons for the use of complementary and alternative medicine respondents were asked the following reasons: their experience has shown that, contrary to conventional medical therapy, complementary medicine therapies safer and have fewer side effects, providers of complementary medicine therapies listeners are better than physicians and the main reason for dissatisfaction with the general physicians of complementary medicine tend to be participants in this study. Results of a study in the UK showed that the use of complementary medicine occurred because of dissatisfaction from general physicians’ service such as lack of communication skills of doctors and inadequate explanations about the nature of the illness and the risk of new drugs (Zollaman & Vickers, 1999). Researcher showed that the most mentioned reasons for complementary medicine are the failure of conventional medicine in the treatment of disease (Mahmoudian, Ebrahim-Babai, & Jafari, 2012). In other studies, various reasons cited by patients for use of complementary medicine such as inefficiency of conventional medicine in the treatment of disease, concerns about the side effects of conventional medicine, weak relationship physician and patient and increase the access to various types of CAM (Furnham & Kirkcaldy, 2006; Vincent & Furnham, 2006). Tehrani-Banihashemi et al., demonstrated that the most common reasons for the selection of complementary and traditional medicine is mentioned useful experience for themselves and others, less risk, less complications of treatment of conventional medicine and failure to adequate respond, respectively (Tehrani-Banihashemi et al., 2008). Recently, Bar-Sela et al., showed that CAM improved significantly outcomes of cancer patients who completed six weekly sessions, regardless of their demographic characteristics (Bar-Sela et al., 2013). Researchers showed that Complementary and alternative medicine use was common in the Diabetes Mellitus (Naja Mousa, Alameddine, Shoaib, Itani, & Mourad, 2014), Eczema (Silverberg, Lee-Wong, & Silverberg, 2014), Duchenne and Becker muscular dystrophies (Zhu et al., 2014) patients. Studies have shown that welcomes people to complementary medicine techniques growing rapidly increasing in developed and developing countries. Use of complementary medicine is increasing because of increase the knowledge of people, many articles about the benefits of alternative medicine along with the increasing number of health care professionals. Furthermore, insurance coverage also plays a role in the increased use of CAM products and services. Planning to educate the public on the proper use of these methods is felt because of the high volume use of complementary medicine.

## References

[ref1] Bar-Sela G, Vorobeichik M, Drawsheh S, Omer A, Goldberg V, Muller E (2013). The Medical Necessity for Medicinal Cannabis: Prospective, Observational Study Evaluating the Treatment in Cancer Patients on Supportive or Palliative Care. Evidence-Based Complementary and Alternative Medicine.

[ref2] Danos S, Visel B, Mashiach T, Mitnik I (2014). The effect of complementary and alternative medicine on quality of life, depression, anxiety, and fatigue levels among cancer patients during active oncology treatment: phase II study. Support Care Cancer, In Press.

[ref3] Furnham A, Kirkcaldy B (2006). The health beliefs and behaviors of orthodox and complementary medicine clients. Br J Clin Psychol.

[ref4] Mahmoudian S. A, Ebrahim-Babai M, Jafari M (2012). The Reasons for and Satisfaction from Using Acupuncture in Isfahan. Journal of Isfahan Medical School.

[ref5] Moradi-Lakeh M, Ramezani M, Ansari H (2008). Factors influencing the use of herbal remedies/medicinal herbs among the general population in Tehran, Iran. Payesh.

[ref6] Naja F, Mousa D, Alameddine M, Shoaib H, Itani L, Mourad Y (2014). Prevalence and correlates of complementary and alternative medicine use among diabetic patients in Beirut, Lebanon: A cross-sectional study. BMC Complement Altern Med.

[ref7] Ock S. M, Choi J. I, Ka Y. S, Lee J, Chun M. S, Huh C. H (2009). The use of complementary and alternative medicine in a general population in South Korea: Results from a national survey in 2006. J Korean Med Sci.

[ref8] Ritchie C. S, Gohmann S. F, McKinney W. P (2005). Does use of CAM for specific health problems increase with reduced access to care?. J Med Syst.

[ref9] Saad B, Azaizeh H, Said O (2005). Tradition and perspectives of Arab Herbal Medicine: A review, Evidence Based complement. Evid Based Complement Alternat Med.

[ref10] Sedighi Z. H, Maftoon F, Moshrefi M (2002). Knowledge and attitude in to the CAM and utilization of these services in the population of Tehran. Payesh.

[ref11] Silverberg J. I, Lee-Wong M, Silverberg N. B (2014). Complementary and alternative medicines and childhood eczema: A US population-based study. Dermatitis.

[ref12] Tehrani-Banihashemi S. A, Asgharifard H, Haghdoost A. A, Barghamdi M, Mohammadhosseini N (2008). The use of Complementary-Alternative Medicine among the general population in Tehran, Iran. Payesh.

[ref13] Vincent C, Furnham A (2006). Why do patients turn to complementary medicine? An empirical study. Br J Clin Psychol.

[ref14] Xue C. C, Zhang A, Lin V, Da Costa C, Story D. F (2007). Complementary and alternative medicine use in Australia: A national population-based survey. J Altern Complement Med.

[ref15] Zhu Y, Romitti P. A, Conway K. M, Andrews J, Liu K, Meaney F. J, Matthews D. J (2014). Complementary and alternative medicine for Duchenne and Becker muscular dystrophies: characteristics of users and caregivers. Pediatr Neurol.

[ref16] Zollaman C, Vickers A (1999). Users and practitioners of complementary medicine. British Medical Journal.

